# Combined score of Y chromosome loss and T-cell infiltration improves UICC based stratification of esophageal adenocarcinoma

**DOI:** 10.3389/fonc.2023.1249172

**Published:** 2023-11-17

**Authors:** Verena Maria Raters, Florian Gebauer, Heike Löser, Wolfgang Schröder, Hans Anton Schlösser, Hans Fuchs, Christiane Bruns, Alexander Quaas, Thomas Zander

**Affiliations:** ^1^ Department I of Internal Medicine, Center for Integrated Oncology Aachen Bonn Cologne Duesseldorf, Gastrointestinal Cancer Group Cologne GCGC, University of Cologne, Cologne, Germany; ^2^ Department of General, Visceral and Cancer Surgery, Gastrointestinal Cancer Group Cologne GCGC, University of Cologne, Cologne, Germany; ^3^ Institute of Pathology, Gastrointestinal Cancer Group Cologne GCGC, University of Cologne, Cologne, Germany

**Keywords:** esophageal adenocarcinoma, Y chromosome loss, CD3 cell infiltration, UICC staging, prognosis

## Abstract

**Background:**

Staging, especially clinical lymph node staging in esophageal adenocarcinoma has only moderate sensitivity and specificity. Therefore, we evaluated combined molecular markers to predict prognosis.

**Patients and methods:**

890 tumor tissue samples were obtained from patients who underwent surgery for esophageal adenocarcinoma with curative intent. These were stained by tissue micro array for 48 markers which are associated with tumorigenesis and correlated with clinical data (TNM-staging, overall survival) by multivariate Cox regression.

**Results:**

Two markers (preserved Y chromosome and high grade of (CD3+) T-cell infiltration) were found to be significantly and independently associated with better overall survival. We formed a score (called CY score) from the two markers. The more markers are positive and thus the higher the score (ranging from 0 to 2), the better the overall survival, independently of UICC. Moreover, we developed a combination score of the UICC and CY score based on cluster analysis. Patients with a UICC stage of III with the presence of both traits (CY=2) can be assigned to a better prognosis group (group II), whereas patients with a UICC stage of I without both traits (CY=0) must be assigned to a worse prognosis group (group II). Therefore, patients in stage I with adverse molecular signature might benefit of multimodal therapy.

**Conclusion:**

In summary, the CY score adds prognostic information to the UICC stage based on tumor biology in esophageal adenocarcinoma and warrants further evaluations in independent clinical cohorts.

## Introduction

Esophageal cancer is a common cancer with a high mortality ranking seventh in terms of incidence and sixth in mortality worldwide (1). In developed countries the incidence of esophageal cancer is rising (2). Whereas very early in the disease endoscopic treatment can lead to cure, surgical resection often combined with chemotherapy or radiochemotherapy was established for locally advanced tumors. Even with these intensive therapeutic regimens the overall survival of patients with esophageal cancer remains poor (3, 4). More recently, also immunotherapy has been introduced in the curative first line setting (5).

Using comprehensive genomic analysis, esophageal cancer has been subdivided in different molecular subtypes, but the prognostic value of these subgroups still remains to be determined (6, 7). In addition, several molecular markers have been evaluated concerning their prognostic value, but none of these has entered clinical routine (preserved Y chromosome (8); HER2 (9); KRas and PIK3CA (10); Integrin alpha-5 (11); ini1, BRM, BRG1 and ARID domain-containing protein 1A (12); Mesothelin (13); GATA-6 (14); XIAP (15); Claudin-18 (16); p53 (17); Mdm2 (18)). In this project we evaluated the potential of combined molecular markers to predict prognosis of esophageal adenocarcinoma.

## Methods

### Patients and tumor samples

893 tumor tissue samples were obtained from patients who underwent surgery for adenocarcinoma of the esophagus with curative intent at the University Hospital Cologne, Germany between 1996 and 2019 and gave informed consent in accordance with The Code of Ethics of the World Medical Association (Declaration of Helsinki) and the local ethics committee (13-091) (**Table 1**). All tumors were classified according to the UICC system of the 7th edition. The tumor tissue samples were stained by tissue micro array (TMA) for 48 markers known to be associated with various aspects of tumorigenesis (proliferation, migration and invasion, immunomodulation, angiogenesis, metabolism, chromatin remodeling, and inflammation). For the following analyses, the 44 markers for which > 30% (n ≥ 270) TMA samples could be evaluated were included: Programmed cell death 1 ligand 1 (PD-L1), T-cell immunoglobulin mucin receptor 3 (TIM-3), Lymphocyte activation gene 3 protein (LAG-3), Receptor tyrosine-protein kinase erbB-2 (HER2), cellular tumor antigen p53, Scavenger receptor class B member 1 (CD36), Carcinoembryonic antigen-related cell adhesion molecule 8 (CD66b), Proliferation marker protein Ki-67, E3 ubiquitin-protein ligase Mdm2, Antigen-presenting major histocompatibility complex class I (MHC-1), High mobility group protein B1 (HMGB1), DNA mismatch repair protein Mlh1, T-cell surface glycoprotein CD3 cell tumor infiltration, Mesothelin, AT-rich interactive domain-containing protein 1A (ARID domain-containing protein 1A), Pre-mRNA-splicing factor ini1, Probable global transcription activator SNF2L2 (BRM), Transcription activator BRG1, Aldo-keto reductase family 1 (AKR1), Claudin-18, Hepatocyte growth factor receptor (MET), Myc proto-oncogene protein, GTPase KRas, Transcription factor GATA-6, Phosphatidylinositol 4,5-bisphosphate 3-kinase catalytic subunit alpha isoform (PIK3CA), preserved Y chromosome, Integrin alpha-5, Integrin beta-1, Integrin beta-4, Phosphatidylinositol 3,4,5-trisphosphate 3-phosphatase and dual-specificity protein phosphatase (PTEN), Fructose-1,6-bisphosphatase 1 (FBP1), Trimethylation of histone H3 lysine 27 (H3K27m3), Ubiquilin-4 (UBQLN4), Tumor-associated calcium signal transducer 2 (TROP-2), F-box/WD repeat-containing protein 7 (FBXW7), G1/S-specific cyclin-D1 (Cyclin D1), Cyclin-dependent kinase inhibitor 2A (CDKN2A/p16), U3 small nucleolar ribonucleoprotein protein IMP3, Cadherin-1 (E-cadherin), Carbonic anhydrase 9, E3 ubiquitin-protein ligase XIAP, Immunoglobulin superfamily DCC subclass member 4 (NOPE) on cancer cells, Hematopoietic progenitor cell antigen CD34 quantity and Gremlin-1 (GREM1) RNA.

**Table 1:** Clinical data of tumor samples.

	*N*	%
Total	893	100
Age (years)		62.7 +/- 10.9 (mean +/- SD)
< 65 years	495	55
≥ 65 years	398	45
Sex		
Male	785	88
Female	108	12
pT		
pT1	161	18
pT2	157	18
pT3	546	61
pT4	29	3
pN		
pN0	363	40
pN1	266	30
pN2	131	15
pN3	133	15
cM		
M0	885	99
M1	8	1
UICC		
I	121	13
II	103	12
III	535	60
IV	134	15
Neoadjuvant therapy		
0=no	298	33
1=yes	595	67
Survival (months)		35.8 +/- 40.8 (mean +/- SD)

In detail, the tumor tissue samples were fixed in 4% buffered formalin at room temperature for at least 24 hours (maximum 72 hours). Tumor tissue microarrays (TMA) were constructed as previously described (19, 20). In brief, tissue cylinders with a diameter of 1.2 mm were punched from selected tumor tissue blocks using an in−house developed semi−automated precision instrument and embedded in empty recipient paraffin blocks. The Paraffin blocks were cut into 4 μm−thick sections, which were transferred onto an adhesive coated slide system. Freshly cut TMA sections were immunostained in one day and in one experiment. Slides were deparaffinized using standard protocols with Dewax (Leica Microsystems, Inc.) and 100% ethanol, and exposed to heat−induced antigen retrieval for 5 min in an autoclave at 121˚C and pH 9 (Tris−EDTA−buffer) or pH 6 (citrate buffer). The TMA slides were stained with the following antibodies (clone, buffer, dilution, manufacturer) or FISH probes (probe name, manufacturer):

AKR1 (EPR14421, EDTA, 1:500, abcam, UK), ARID domain-containing protein 1A (EPR13501, EDTA, 1:1000, abcam, UK), BRM (D9E8B, EDTA, 1:50, Cell Signaling Technology, MA, USA), BRG1 (EPNCIR111A, EDTA, 1:300, abcam, UK), Carbonic anhydrase IX (EPR4151, EDTA, 1:100, abcam, UK), CD3 (SP7, citrate, 1:50, Thermo Fisher Scientific, MA, USA), CD34 quantity (QB End10, citrate, 1:700, Cell Marque, CA, USA), CD36 (D8L9T, citrate, 1:200, Cell Signaling Technology, MA, USA), CD66b (G10F5, EDTA, 1:200, Novus Biologicals, CO, USA), CDKN2A/p16 (ZytoLight ^®^ SPEC CDKN2A/CEN 9 Dual Color Probe, Zytomed, Germany), Claudin-18 (ERP19202, EDTA, 1:200, abcam, UK), c-Myc (Y69, citrate, 1:100, abcam, UK), Cyclin D1 (ZM178, citrate, 1:400, Zeta Corporation, CA, USA), E-Cadherin (M3612, EDTA, 1:50, Dako, CA, USA), FBP1 (EPR4619, EDTA, 1:100, abcam, UK), FBXW7 (SP237, EDTA, 1:500, abcam, UK), GATA-6 (GATA6-20-GR Probe, Empire Genomics, NY, USA), Grem1 (ab22138, EDTA, 1:400, abcam, UK), H3K27m3 (C36B11, EDTA, 1:100, Cell Signaling Technology, MA, USA), HER2 (4b5, EDTA, not diluted, Roche, Switzerland), HMGB1 (D3E5, EDTA, 1:500, Cell Signaling Technology, MA, USA), IMP3 (M3626, EDTA, 1:100, Agilent, Dako, CA, USA), ini1 (BCIR1, EDTA, 1:50, Zytomed Systems, Germany), Integrin alpha-5 (EPR7854, EDTA, 1:300, abcam, UK), Integrin beta-1 (A4, EDTA, 1:100, Santa Cruz Biotechnology, Germany), Integrin beta-4 (D8P6C, EDTA, 1:100, Cell Signaling Technology, MA, USA), Ki-67 (SP6, EDTA, 1:100, Cell Marque, CA, USA), KRas (9.13, citrate, 1:100, Thermo Fisher Scientific, MA, USA), LAG-3 (D2G40, EDTA, 1:300, Cell Signaling Technology, MA, USA), Mdm2 (Ab-1/IF2, EDTA, 1:50, Calbiochem, NJ, USA), Mesothelin (5B2, EDTA; 1:50, Novocastra, Switzerland), MET (SP44, EDTA, not diluted, Roche, Switzerland), MHC-1 (EPR1394Y, citrate, 1:300, abcam, UK), Mlh1 (M1, EDTA, not diluted, Roche, Switzerland), NOPE-Ca (RNAscope^®^ 2.5 LS Probe- Hs-IGDCC4, ACD, CA, USA), p53 (DO-7, citrate, 1:800, Dako, CA, USA), PD-L1 (E1L3N, EDTA, 1:400, Cell Signaling Technology, MA, USA), PIK3CA (6D9, EDTA, 1:1000, abnova, Taiwan), PTEN (138 G 6, EDTA, 1:300, Cell Signaling Technology, MA, USA), TIM-3 (D5D5R, EDTA, 1:100, Cell Signaling Technology, MA, USA), TROP-2 (ERP20043, EDTA, 1:1000, abcam, UK), UBQLN4 (RNAscope^®^ 2.5 LS Probe- Hs-UBQLN4, ACD, CA, USA), XIAP (ab21278, citrate, 1:1000, abcam, UK), preserved Y chromosome (long and short arm) (Vysis LSI SRY Spectrum Orange Probe and Vysis CEP Y (DYZ1) Spectrum Green Probe, Abbott Molecular, Germany).

The staining was evaluated and quantified by a pathologist and the open-source software QuPath and classified as negative or positive staining for the marker. The two evaluation strategies were compared with very high concordance of results. In the case of discordance, the pathologist determined the division into negative/positive.

Concerning CD3 cell infiltration into the tumor, we used two different evaluation strategies: a semiquantitative method in which a pathologist (AQ) estimated the extent of T lymphocytes in the stroma and divided the extent into the two groups “low” and “high” and a digital method using the freely available software QuPath. QuPath standardized the amount of CD3 positive cells in the tissue to 1 mm^2^ in absolute numbers, the median was then taken to divide into the two groups “low” and “high”. This was then compared to the primary semiquantitative assessment with very high concordance of results. In the case of discordance, the pathologist determined the division into “low” and “high”.

Clinical data (especially age, sex, survival time, survival status, last follow-up, date of surgery, whether neoadjuvant therapy had been given, state at the time of surgery (pathological tumor extent (y)pT, pathological nodal state (y)pN, clinical metastasis state (cM) and UICC-stage) were collected prospectively according to a standardized protocol.

### Statistical analysis

For statistical analysis, IBM SPSS Statistics for Windows (Version 27) was used.

First, univariate Cox regression was used to test the correlation of each marker with survival time. Significant markers with a *p*-value (according to Bonferroni correction for multiple comparison) ≤ 0.001 were tested in multivariate Cox regression. A score (called CY score) was formed from the two remaining markers (detection of preserved Y chromosome and high grade of CD3 cell infiltration). A score value of 0 indicates that no marker is positive (loss of Y chromosome and low CD3 cell infiltration), 1 indicates that one marker is positive (either preserved Y chromosome or high CD3 cell infiltration), and 2 indicates that both markers are positive (preserved Y chromosome and high grade of CD3 cell infiltration). The calculation of the score was possible in 620 cases since there staining for both markers was available. To test the correlation of each marker (detection of preserved Y chromosome and CD3 cell infiltration) as well as of the CY score with survival time, a Kaplan-Meier curve was calculated comparing groups using the log-rank test. In addition, a multivariate Cox regression including UICC was calculated. In the next step, the correlation of the markers (detection of preserved Y chromosome and CD3 cell infiltration) and CY score with (y)pT, (y)pN and (y)UICC were tested by calculating the Spearman coefficient and cross tabulation, respectively.

To check for CY score validity in subgroups, Cox regression of the CY score was calculated for patients with a lower versus higher UICC stage (UICC stage 1 and 2 versus 3 and 4), for patients having undergone neoadjuvant chemotherapy or not, for younger versus older patients (< versus ≥ 65 years) and for male versus female patients.

Furthermore, the UICC stage was combined with the CY score. Therefore, the strata of the UICC stage were substratified based on the three groups of the CY score. Using cluster analysis with Ward’s method and squared Euclidean distance as the proximity measure, these were reassembled into new groups of at least two substrata based on their similarity with respect to 0.5-, 1-, 1.5-, 2-, 2.5-, 3-, 3.5-, 4-, 4.5-, 5-, 5.5-, 6-, 6.5- and 7-year survival. To test the discriminatory power of the new CY-UICC score, a Kaplan-Meier curve was calculated comparing groups using the log-rank test.

All tests were two-sided; *p* values < 0.05 were considered statistically significant. Significance was marked as follows: * for *p* ≤ 0.05, ** for *p* ≤ 0.01 and *** for *p* ≤ 0.001.

## Results

### Preserved Y chromosome and CD3 cell infiltration in the tumor are the most significant markers correlating with longer overall survival

As a first step, we examined which of the 44 markers correlated with prolonged overall survival (**Table 2**).

**Table 2:** Marker: analyzable TMAs (n), mean of marker expression between 0 (negative) and 1 (positive), p value and hazard ratio of univariate Cox regression of the marker and overall survival.

Marker	N	mean	*p*	hazard ratio
AKR1	448	0.91	0.09	0.72
ARID1A	806	0.9	0.73	0.96
BRG1	680	0.97	0.97	0.77
BRM	693	0.9	0.23	1.25
Carbonic anhydrase IX	330	0.5	0.13	0.81
CD3 cell tumor infiltration	775	0.35	0.00003	0.64
CD34	331	0.4	0.12	0.8
CD36	389	0.42	0.001	0.66
CD66b	587	0.48	0.79	0.97
CDKN2A/p16	416	0.3	0.023	1.16
Claudin-18	440	0.18	0.56	1.1
c-Myc	463	0.18	0.47	1.11
Cyclin D1	549	0.57	0.98	0.988
E-Cadherin	331	0.96	0.55	0.82
FBP1	525	0.82	0.006	0.67
FBXW7	511	0.93	0.38	0.82
GATA-6	450	0.11	0.21	0.78
Grem1	291	0.9	0.54	0.87
H3K27m3	382	0.87	0.42	0.86
Her2	766	0.08	0.007	0.57
HMGB1	499	0.77	0.13	0.82
IMP3	330	0.76	0.007	1.59
ini1	498	1.0	0.16	0.24
Integrin alpha-5	568	0.15	0.14	1.22
Integrin beta-1	581	0.2	0.02	1.36
Integrin beta-4	400	0.79	0.008	0.67
Ki67	389	0.54	0.76	1.04
KRas	477	0.19	0.08	1.29
Lag3	398	0.36	0.14	0.82
Mdm2	386	0.05	0.82	1.07
Mesothelin	429	0.38	0.18	1.19
MET	447	0.09	0.63	1.11
MHC-1	388	0.96	0.84	1.06
Mlh1	807	0.96	0.57	1.3
NOPE-Ca	333	0.08	0.25	1.31
p53	775	0.8	0.75	0.96
PD-L1	451	0.14	0.56	0.9
PIK3CA	416	0.06	0.82	1.06
PTEN	564	0.92	0.03	0.66
TIM3	448	0.54	0.7	0.95
TROP-2	546	0.89	0.97	1.0
UBQLN4	418	0.77	0.98	1.0
XIAP	335	0.91	0.55	1.17
preserved Y chromosome	681	0.41	0.001	0.68

Then, a multivariate Cox regression was performed on the markers that correlated significantly with prolonged overall survival. The markers high CD3 cell infiltration into the tumor (*p* = 0.003, HR 0.69) and preserved Y chromosome (*p* = 0.005, HR 0.72) were found to stay significant and remained significant after adding UICC to the multivariate survival analysis.

### A score of preserved Y chromosome and high CD3 cell infiltration in the tumor correlates with better overall survival

**Figure 1:**
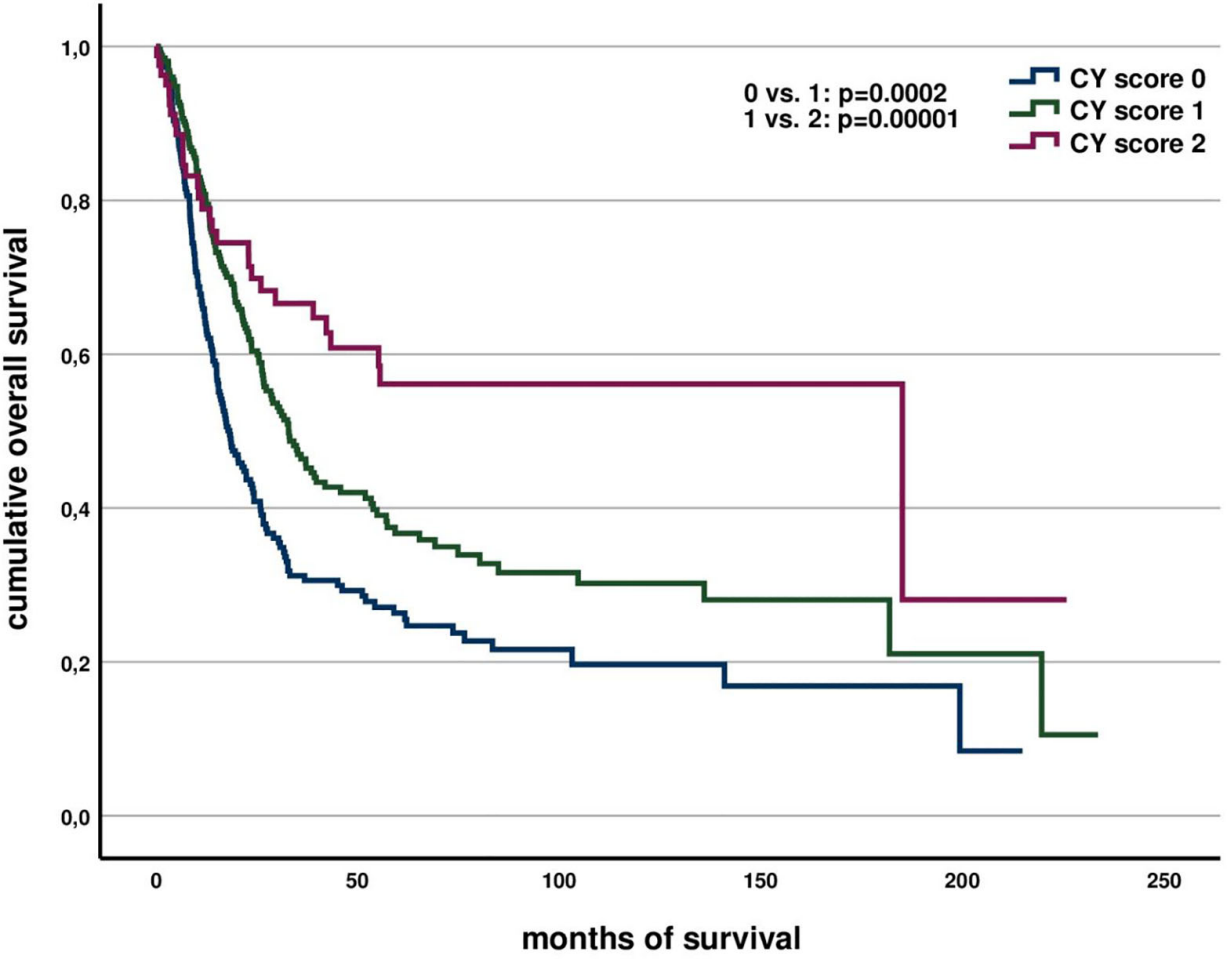
Association of CY score with overall survival.

A score was formed from these two markers. Here, 0 corresponds to no positive marker (loss of Y chromosome and low infiltration of CD3 cells), 1 to one positive marker, and 2 to both markers being positive (Y chromosome preserved and high CD3-cell infiltration into the tumor). Higher CY scores were significantly associated with better survival (CY=0 vs. 1 *p*=0.0002, CY=1 vs. 2 *p*=0.00001) (**Figure 1**). This was also true when controlling for UICC stage in a multivariate COX regression (*p*=0.00007).

### Preserved Y chromosome and high CD3 cell infiltration in the tumor are correlated with lower N staging in treatment-naïve patients

Correlation of the individual markers and the CY score with other clinical parameters demonstrated an association of high CD3 cell tumor infiltration and preserved Y chromosome with longer survival, a low pN and a low UICC. A high CY score additionally correlates with a smaller pT. All these associations are only to be seen in treatment-naïve patients (see **Table 3**). Histologic grading, treatment effect (Cologne regression score), lymphovascular invasion and margin status did not show a significant association with the CY score.

**Table 3:** Association of markers with clinical data.

	high CD3 expression	preserved Y chromosome	CY score
survival	*p*=0.001	*p*=0.003	*p*=0.00007
pT + ypT	ns	p=0.03	*p*=0.005
pT	ns	*p*=0.003	*p*=0.002
ypT	ns	ns	ns
pN + ypN	*p*=0.03	*p*=0.009	*p*=0.00001
pN	*p=*0.03	*p*=0.00006	*p*=0.00001
ypN	*p*=0.04	ns	ns (*p*=0.06)
UICC + yUICC	*p=*0.02	*p*=0.002	*p*=0.00005
UICC	ns (*p*=0.07)	*p*=0.0006	*p*=0.0001
yUICC	ns	ns	ns

ns, non-significant.

### The CY score is prognostic for all subgroups but for female patients

Concerning the validity of the CY score for prognosis of survival in subgroups, it is valid for patients with low (*p*=0.039) versus high (*p*=0.00016) UICC stage, for patients having undergone neoadjuvant therapy (*p*=0.002) or not (*p*=0.0003) and for younger (<65 years) (*p*=0.001) and older patients (*p*=0.00006). Concerning female patients, a subgroup analysis was not possible since in the analyzed cohort the score had been able to be calculated in too few women (*n*=28).

### The CY score in combination with the UICC stage identifies patients with a better or worse prognosis based on tumor biology

**Figure 2:**
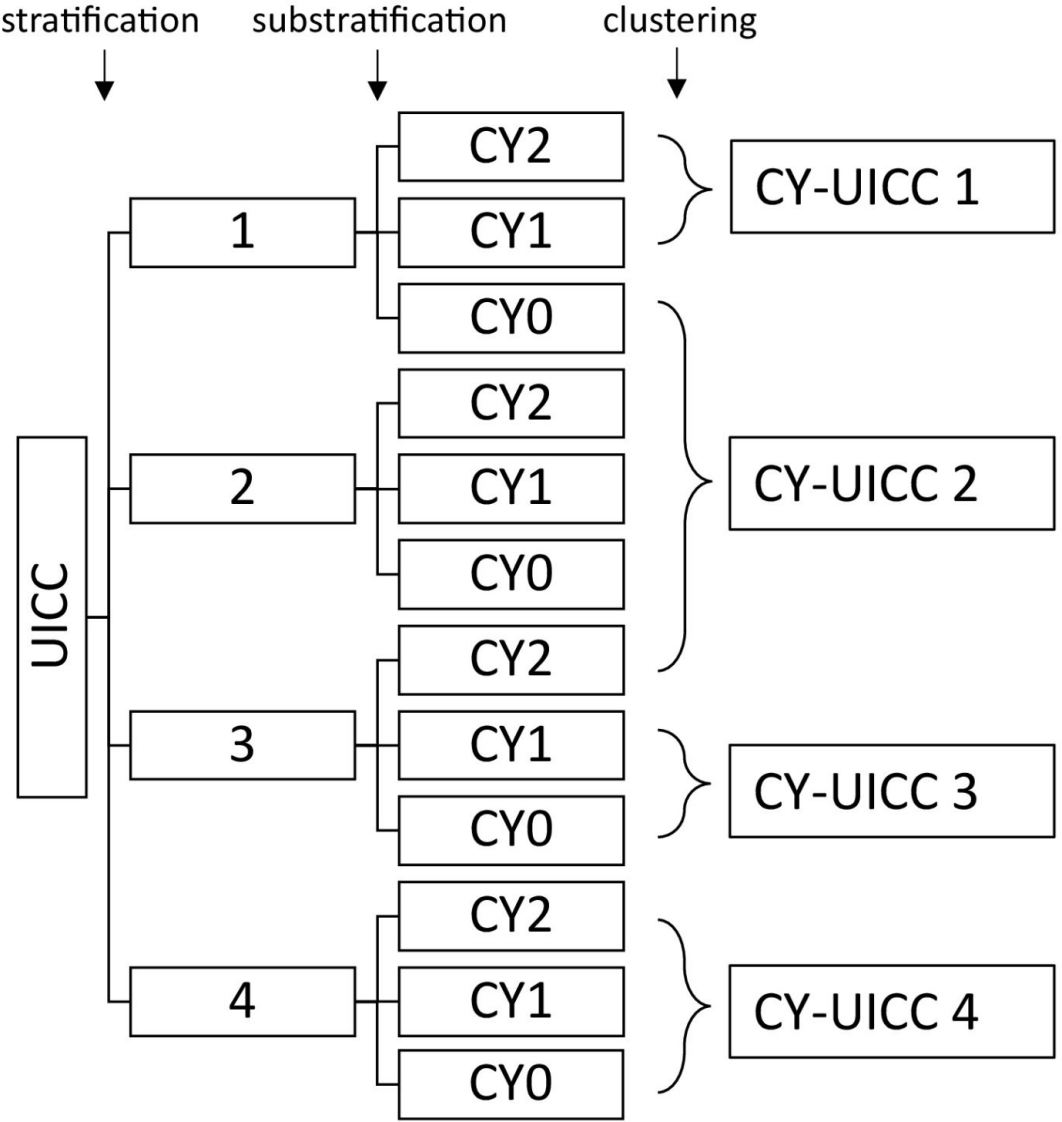
Clustering groups to form the CY-UICC score.

Substratifying the UICC stage using the three groups of the CY score and clustering these substrata by survival over time results in a new combination score. This newly formed combination score (CY-UICC score) is composed according to **Figure 2**.

**Figure 3:**
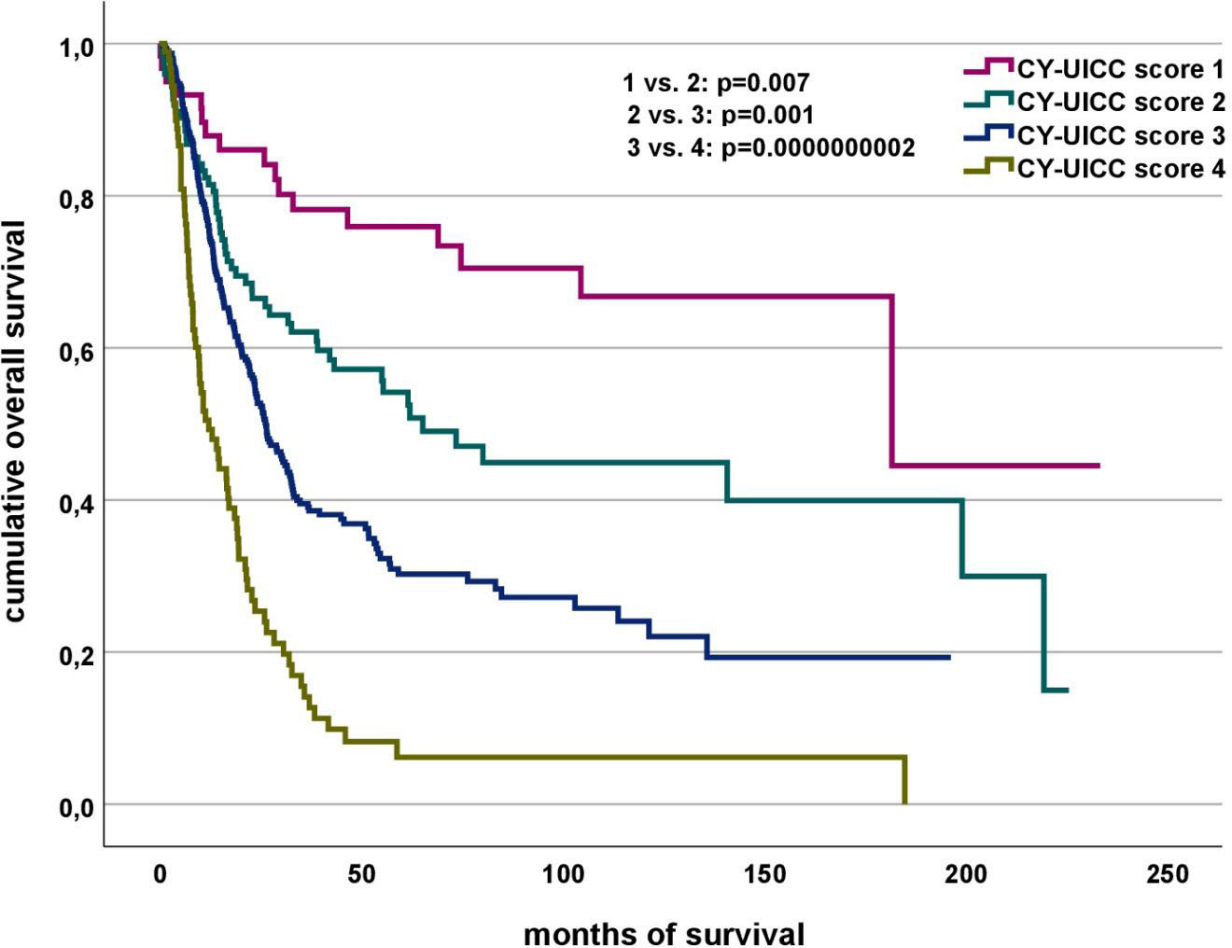
Association of CY-UICC score and overall survival.

The CY-UICC score significantly separates four different prognostic groups (group 1 vs. 2 *p*= 0.007, 2 vs. 3 *p*=0.001, 3 vs. 4 *p*=0.000000002) (see **Figure 3**). Median overall survival in the groups is 70.2 months (CY-UICC score 1), 42.5 months (CY-UICC score 2), 30.0 months (CY-UICC score 3) and 16.1 months (CY-UICC score 4), respectively.

**Figure 4:**
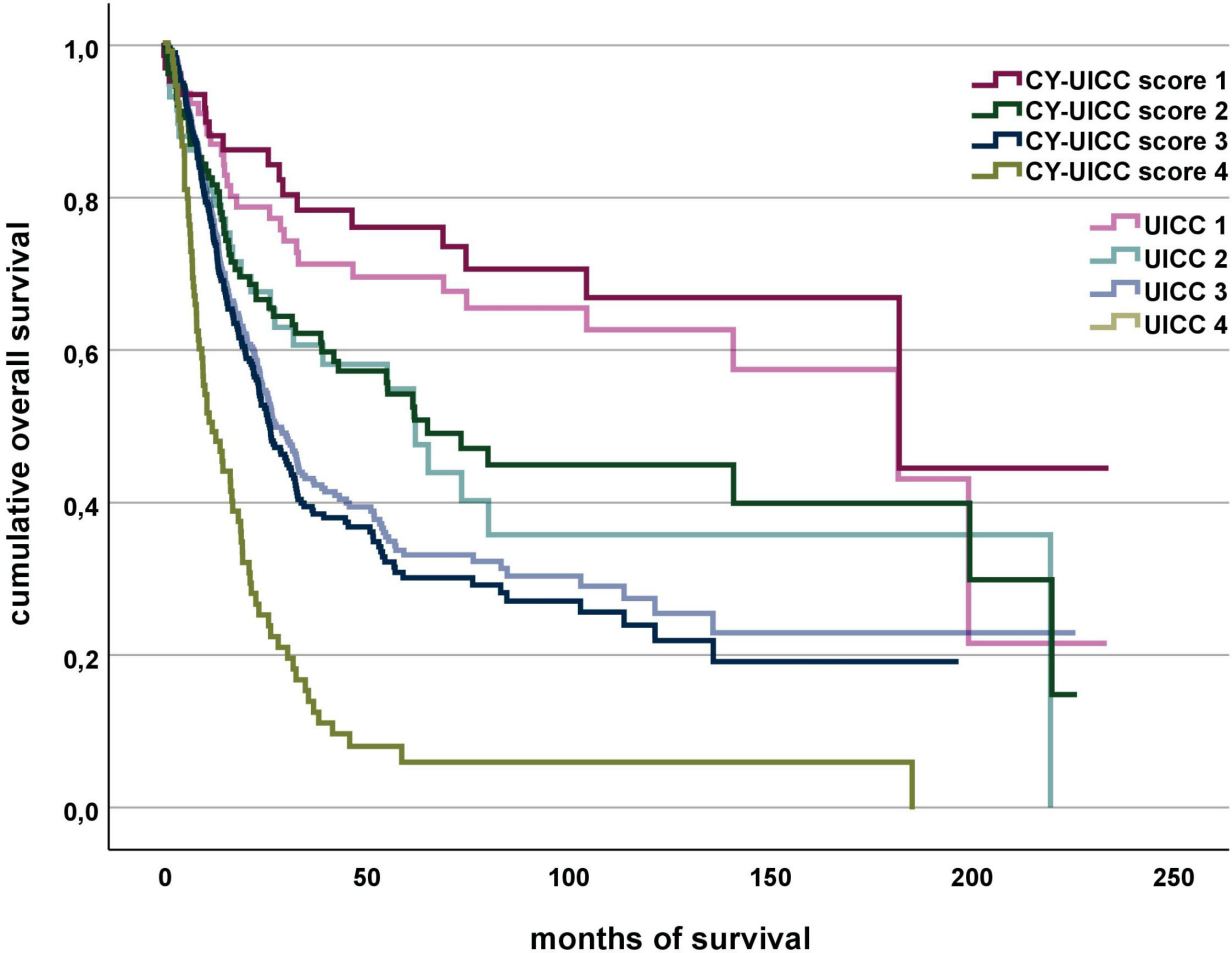
Association of CY-UICC score vs. UICC and overall survival. The groups of CY-UICC score 4 and UICC 4 are identical.

Clustering of the CY-UICC score groups shows that considering the CY score, patients with a UICC stage of 3 with the presence of both traits (CY=2) can be assigned to a better prognosis group (group 2), whereas patients with a UICC stage of 1 without both traits (CY=0) must be assigned to a worse prognosis group (group 2) (see **Figure 2**). **Figure 4** compares overall survival of the newly developed CY-UICC score and UICC.

## Discussion

We have shown that a preserved Y chromosome and high CD3 cell infiltration in the tumor are important markers for prolonged overall survival in esophageal adenocarcinoma.

Y chromosome loss (LoY) is associated with aging in men and occurs more frequently in men who smoke (21). LoY is associated with a higher risk for developing non-hematological cancer in general (22). Specifically, we have shown that LoY is a common phenomenon in esophageal adenocarcinoma and is associated with shorter overall survival (8). The functional effect leading to decreased survival in esophageal cancer has not been fully elucidated until yet. It might be a marker for general chromosomal instability and is detectable already in precursing Barrett´s esophagus (23). Furthermore, LoY was shown to be associated with an epigenetic phenotype with methylation of genes being involved in cell proliferation and cell cycle regulation (21). Additionally, the loss of function of genes of the nonrecombinant region of Y were shown to play a role in cell cycle regulation and behave as dosage-sensitive tumor suppressors (24).

Tumor infiltrating lymphocytes (TIL) are involved in immune editing of tumor cells. By elimination of (immunogenic) tumor cells, they reduce tumor growth and at the same time support the eventual outgrowth of less immunogenic tumor cells by selection pressure (25, 26).

Overall, high lymphocyte infiltration of the tumor is associated with longer survival. CD3 cell tumor infiltration is known as good prognostic factor concerning survival in ductal breast cancer (27), non-small cell lung cancer (NSCLC) (28), intestinal-type gastric cancer (29), hepatocellular carcinoma (HCC) (30), gastrointestinal stromal tumor (GIST) (31), ovarian cancer (32), bladder cancer (33), oral squamous cell carcinoma (34) and nasopharyngeal carcinoma (35). For colon cancer, it was even shown that immune cell density in the tumor was of more prognostic importance than UICC classification (36).

In this paper, we have shown that using the CY score adds prognostic information to the UICC stage, most probably by resembling tumor biology (especially immunogenicity and genetic stability). Limitations of the study are its monocentric and retrospective character, a strength its large sample size for this comparably rare type of cancer. Staging, especially clinical lymph node staging is difficult, as sensitivity and specificity of lymph node detection in CT scans and endoscopic ultrasound have low sensitivity (52 - 81%) and specificity (73 - 87%) (37–39). As guidelines recommend multimodal therapy for patients with esophageal cancer in UICC stage II/III, lymph node staging is performed even though diagnostic techniques have limitations. Here, we show that patients in UICC stage I with adverse molecular signature (CY score 0) clinically behave like UICC stage II patients and might benefit from multimodal therapy. UICC stage III patients with a favorable tumor biology (CY score 2) have a better prognosis compared to UICC stage III patients with a less favorable tumor biology. UICC stage IV patients with a good tumor biology (CY score 2) are a very rare event (CY-UICC=2 *n=*8 vs. CYUICC=1 *n=*46 and CYUICC=0 *n=*48) and in these cases the protective effect of the good tumor biology probably cannot outweigh the adverse stage IV cancer features.

In summary, the CY score adds prognostic information to the UICC stage and warrants further evaluations in independent clinical cohorts.

## Data availability statement

The raw data supporting the conclusions of this article will be made available by the authors, without undue reservation.

## Ethics statement

The studies involving humans were approved by ethics committee of the University Hospital of Cologne (13-091). The studies were conducted in accordance with the local legislation and institutional requirements. The participants provided their written informed consent to participate in this study.

## Author contributions

VR: Conceptualization, Methodology, Formal analysis, Writing - original Draft, FG: Data curation, HL: Investigation, WS: Resources, HS: Resources, HF: Resources, CB: Resources, AQ: Conceptualization, Supervision, Investigation, Writing- Reviewing and Editing, TZ: Conceptualization, Supervision, Writing- Reviewing and Editing. All authors contributed to the article and approved the submitted version.
